# OCR-Stats: Robust estimation and statistical testing of mitochondrial respiration activities using Seahorse XF Analyzer

**DOI:** 10.1371/journal.pone.0199938

**Published:** 2018-07-11

**Authors:** Vicente A. Yépez, Laura S. Kremer, Arcangela Iuso, Mirjana Gusic, Robert Kopajtich, Eliška Koňaříková, Agnieszka Nadel, Leonhard Wachutka, Holger Prokisch, Julien Gagneur

**Affiliations:** 1 Department of Informatics, Technical University of Munich, Garching, Germany; 2 Quantitative Biosciences Munich, Gene Center, Department of Biochemistry, Ludwig-Maximilians Universität München, Munich, Germany; 3 Institute of Human Genetics, Helmholtz Zentrum München, Neuherberg, Germany; 4 Institute of Human Genetics, Klinikum Rechts der Isar, Technical University of Munich, Munich, Germany; University of Alabama at Birmingham, UNITED STATES

## Abstract

The accurate quantification of cellular and mitochondrial bioenergetic activity is of great interest in medicine and biology. Mitochondrial stress tests performed with Seahorse Bioscience XF Analyzers allow the estimation of different bioenergetic measures by monitoring the oxygen consumption rates (OCR) of living cells in multi-well plates. However, studies of the statistical best practices for determining aggregated OCR measurements and comparisons have been lacking. Therefore, to understand how OCR behaves across different biological samples, wells, and plates, we performed mitochondrial stress tests in 126 96-well plates involving 203 fibroblast cell lines. We show that the noise of OCR is multiplicative, that outlier data points can concern individual measurements or all measurements of a well, and that the inter-plate variation is greater than the intra-plate variation. Based on these insights, we developed a novel statistical method, OCR-Stats, that: i) robustly estimates OCR levels modeling multiplicative noise and automatically identifying outlier data points and outlier wells; and ii) performs statistical testing between samples, taking into account the different magnitudes of the between- and within-plate variations. This led to a significant reduction of the coefficient of variation across plates of basal respiration by 45% and of maximal respiration by 29%. Moreover, using positive and negative controls, we show that our statistical test outperforms the existing methods, which suffer from an excess of either false positives (within-plate methods), or false negatives (between-plate methods). Altogether, this study provides statistical good practices to support experimentalists in designing, analyzing, testing, and reporting the results of mitochondrial stress tests using this high throughput platform.

## Introduction

Mitochondria are double-membrane-enclosed, ubiquitous, maternally inherited organelles present in most eukaryotic cells [1]. They are known as the powerhouse of the cell [2,3] due to their pivotal function in the cellular energy supply where adenosine triphosphate (ATP) is generated by the mitochondrial respiratory chain in a process referred to as oxidative phosphorylation. Furthermore, mitochondria are involved in regulating reactive oxygen species [4], apoptosis [2], amino acid synthesis [5,6], cell proliferation [6], cell signaling [7], and in the regulation of innate and adaptive immunity [8]. A decline in mitochondrial function, reflected by a diminished electron transport chain activity, is related to many human diseases ranging from rare genetic disorders [9] to common ones such as cancer [7,10], diabetes [11], neurodegeneration [12], and aging [3]. One of the most informative tests of mitochondrial function is the quantification of cellular respiration, since it directly reflects electron transport chain impairment [9] and depends on many sequential reactions from glycolysis to oxidative phosphorylation [13]. One of the last steps of cellular respiration is the oxidation of cytochrome c in complex IV, which reduces oxygen to form water. Therefore, the estimations of oxygen consumption rates (OCR) expressed in pmol/min enable drawing conclusions about the ability to synthesize ATP and about mitochondrial function, even more than the measurements of intermediates (such as ATP or nicotinamide adenine dinucleotide NADH) and potentials [14,15].

OCR was classically measured using a Clark-type electrode, which is time-consuming, limited to whole cells in suspension and high yield, and does not allow the automated injection of compounds [15]. In addition, experimentation with isolated mitochondria is ineffective because the cellular regulation of mitochondrial function is removed during isolation [16]. In the last few years, a new technology that calculates O_2_ concentrations from fluorescence [17] in a microplate assay format has been developed by the company Seahorse Bioscience (now part of Agilent Technologies) [18]. It allows simultaneous real-time measurements of both OCR and extracellular acidification rate (ECAR) in multiple cell lines and conditions, reducing the amount of required sample material and increasing the throughput [18,19].

**Fig 1:**
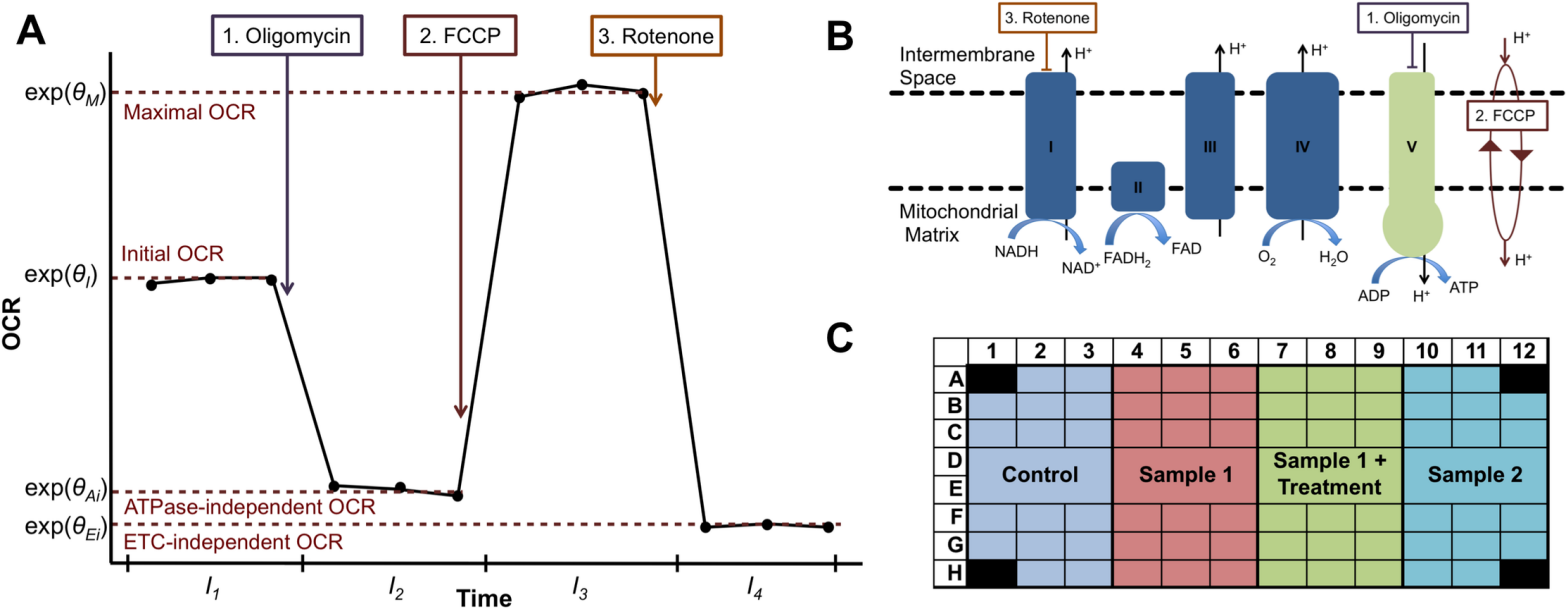
Principle of the mitochondrial stress test assay. (**A**) Cartoon illustration of OCR levels (y-axis) versus time (x-axis). Injection of the three compounds oligomycin, FCCP, and rotenone delimits four time intervals within each of which OCR is roughly constant. (**B**) Targets of each compound in the electron transport chain. (**C**) Typical layout of a mitochondrial stress test 96-well plate.

Typically, OCR and ECAR are measured using the Seahorse XF Analyzer in 96-well (or 24-well) plates at multiple time steps under three consecutive treatments (Fig 1), in a procedure known as a mitochondrial stress test [20]. Under basal conditions, complexes I–IV exploit energy derived from electron transport to pump protons across the inner mitochondrial membrane. The proton gradient generated in this manner is subsequently harnessed by complex V to generate ATP. The blockage of the proton translocation through complex V by injecting oligomycin represses ATP production and prevents the electron transport throughout complexes I–IV due to the unexploited gradient, thus, generating ATP-ase independent OCR only (Fig 1A and 1B). The administration of carbonyl cyanide-4-(trifluoromethoxy)phenylhydrazone (FCCP), an ionophor, subsequently dissipates the gradient uncoupling electron transport from complex V activity and increasing oxygen consumption to a maximum level (Fig 1A and 1B). Finally, mitochondrial respiration is completely halted using rotenone, a complex I inhibitor. There is still some remaining oxygen consumption that is independent from electron transport chain activity (Fig 1A and 1B). This approach is label-free and non-destructive, so the cells can be retained and used for further assays [21].

OCR differences in the natural scale between the various stages of this procedure lead to the estimation of six different bioenergetic measures: basal respiration, proton leak, non-mitochondrial respiration, ATP-linked respiration, spare respiratory capacity, and maximal respiration [15,19] (Table 1). An increase in proton leak and a decrease in basal or maximal respiration are indicators of mitochondrial dysfunction [15]. In addition, ATP-linked respiration, basal respiration, and spare capacity change in response to ATP demand, which is not necessarily mitochondrion-related as it may be the consequence of the dysregulation of any cellular process altering general cellular energy demand. Then, these bioenergetic measures are typically used to test two samples or conditions against each other.

**Table 1:** OCR ratios, abbreviations, definitions, metrics, and analogous definitions.

OCR ratios	Abbr.	Definition	Metrics	Analogous in literature
ETC-dependent OCR proportion	E/I-proportion	Proportion of OCR in the ETC with respect to the initial OCR	$\frac{{OCR}_{1}-{OCR}_{4}}{{OCR}_{1}}=1-exp(\theta_{Ei}-\theta_{I})$	Basal respiration: *OCR*_1_–*OCR*_4_
ATPase-dependent OCR proportion	A/I-proportion	Proportion of OCR driven from ATPase proton pumping with respect to the initial OCR	$\frac{{OCR}_{1}-{OCR}_{2}}{{OCR}_{1}}=1-exp(\theta_{Ai}-\theta_{I})$	ATP-linked respiration: *OCR*_1_–*OCR*_2_
ETC-dependent proportion of ATPase-independent OCR	E/Ai-proportion	Proportion of OCR in the ETC, but not driven from ATPase proton pumping, with respect to all non ATPase driven OCR	$\frac{{OCR}_{2}-{OCR}_{4}}{{OCR}_{2}}=1-exp(\theta_{Ei}-\theta_{Ai})$	Proton leak: *OCR*_2_–*OCR*_4_
Maximal over initial OCR fold change	M/I-fold change	Ratio between maximal OCR and initial OCR	$\frac{{OCR}_{3}}{{OCR}_{1}}=exp(\theta_{M}-\theta_{I})$	Spare respiratory capacity: *OCR*_3_–*OCR*_1_
Maximal over ETC-independent OCR fold change	M/Ei-fold change	Ratio between maximal OCR and non-ETC driven OCR	$\frac{{OCR}_{3}}{{OCR}_{4}}=exp(\theta_{M}-\theta_{Ei})$	Maximal respiration: *OCR*_3_–*OCR*_4_
Not defined as a ratio	*NA*	*NA*	*NA*	Non-mitochondrial respiration: *OCR*_4_

Proposed definitions for cellular bioenergetics based on ratios, their abbreviations, equations to compute them and analogous measures used in the literature. *OCR*_*i*_ and *θ*_*i*_ correspond to the expected OCR value on time interval *i* in the natural and logarithmic scale, respectively (Fig 1A).

The existing literature describing the Seahorse technology addresses experimental aspects regarding sample preparation [22,23], the number of cells to seed [23,24], and compound concentration in different organisms [13,22,25]. However, studies regarding statistical best practices for determining OCR levels and testing them against others are lacking. The sole definition of bioenergetic measures varies between authors, as well as the number of time points in each interval (usually three time points, but in some cases one [26], two [27], or four or more [11]), and whether differences [6,13,28], ratios [12,29], or both [24,25] should be computed. Consequently, the comparison of results across studies is difficult. Moreover, often, statistical power analyses for experimental design are not provided. The differences in OCR between biological samples (e.g. patient vs. control, or gene knockout vs. WT) can be as low as 12%–30% [30–32]. Therefore, to design experiments with appropriate power to significantly detect such differences, it is important to know the source and amplitude of the variation within each sample, and to reduce it as much as possible.

We performed and analyzed a large dataset of 126 experiments in 96-well plate format involving 203 different fibroblast cell lines, out of which 26 were seeded in more than one plate (S1 Table). The large number of between-plate and within-plate replicates allowed us to statistically characterize the nature and magnitude of systematic and random variations in these data. We developed a statistical procedure called OCR-Stats, to extract robust and accurate oxygen consumption rates for each well, which translates into robust summarized values of the multiple replicates within and between plates. The OCR-Stats algorithm includes automatic outlier identification and controls for well and plate-interval effects, which led to a significant increase in accuracy over state-of-the-art methods.

Systematic and random variations were found to be multiplicative. This motivated us to establish bioenergetic measures based on differences in the logarithmic scale that translate into ratios and proportions in the natural scale: ETC-dependent OC proportion, ATPase-dependent OC proportion, ETC-dependent proportion of ATPase-independent OC, and maximal over initial OC fold change (Table 1).

Using an automatic outlier detection approach, we provide estimators for each instance and show empirically that they are normally distributed. This permitted the use of linear regression models for assessing the statistical significance of bioenergetic measure comparisons between two biological samples. Using positive and negative controls from individuals known to have mitochondrial respiratory defects, we show that OCR-Stats outperforms the currently used statistical tests, which suffer from an excess of either false positives (within-plate methods) or false negatives (between-plate methods).

Furthermore, our study provides experimental design guidance by i) showing that between-plate variation largely dominates within-plate variation, implying that it is important to seed the same cell lines in multiple plates, and ii) providing estimates of variances within and between plates for each bioenergetic measure allowing for statistical power computations. A free and pose source implementation of OCR-Stats in the statistical language R is provided at github.com/gagneurlab/OCR-Stats.

## Results

### Experimental design and raw data

We measured the OCR, the ECAR, and the cell number of 203 dermal fibroblast cultures derived from patients suspected to suffer from rare mitochondrial diseases and control cells from healthy donors (normal human dermal fibroblasts: NHDF, Materials and methods, S1 Table). These were assayed in 126 plates, all using the same protocol (Materials and methods). Twenty-six cell lines were grown independently and were measured in multiple plates. We will refer to these growth replicates as different biological samples. The NHDF cell line was seeded in all the plates for the assessment of potential systematic plate effects. The corners of each plate were left as blank, that is, filled with media but not with cells, to control for changes in temperature [22]. One common layout of a plate is depicted in Fig 1C, showing how each biological sample is present in many well replicates. We seeded between 3 and 7 biological samples per plate (median = 4). This variation reflects typical set-ups of experiments in a lab performed over multiple years. Then, we used the standard mitochondrial stress test assay [20] leading to four time intervals, with three time points each, denoted by Int_1_ (before adding any treatment), Int_2_ (after oligomycin), Int_3_ (after FCCP), and Int_4_ (after rotenone) (Fig 1A). In addition, we flagged wells that did not react as expected to the treatments and discarded them from the statistical analysis (Materials and methods).

### Variations between replicates within plates

**Fig 2:**
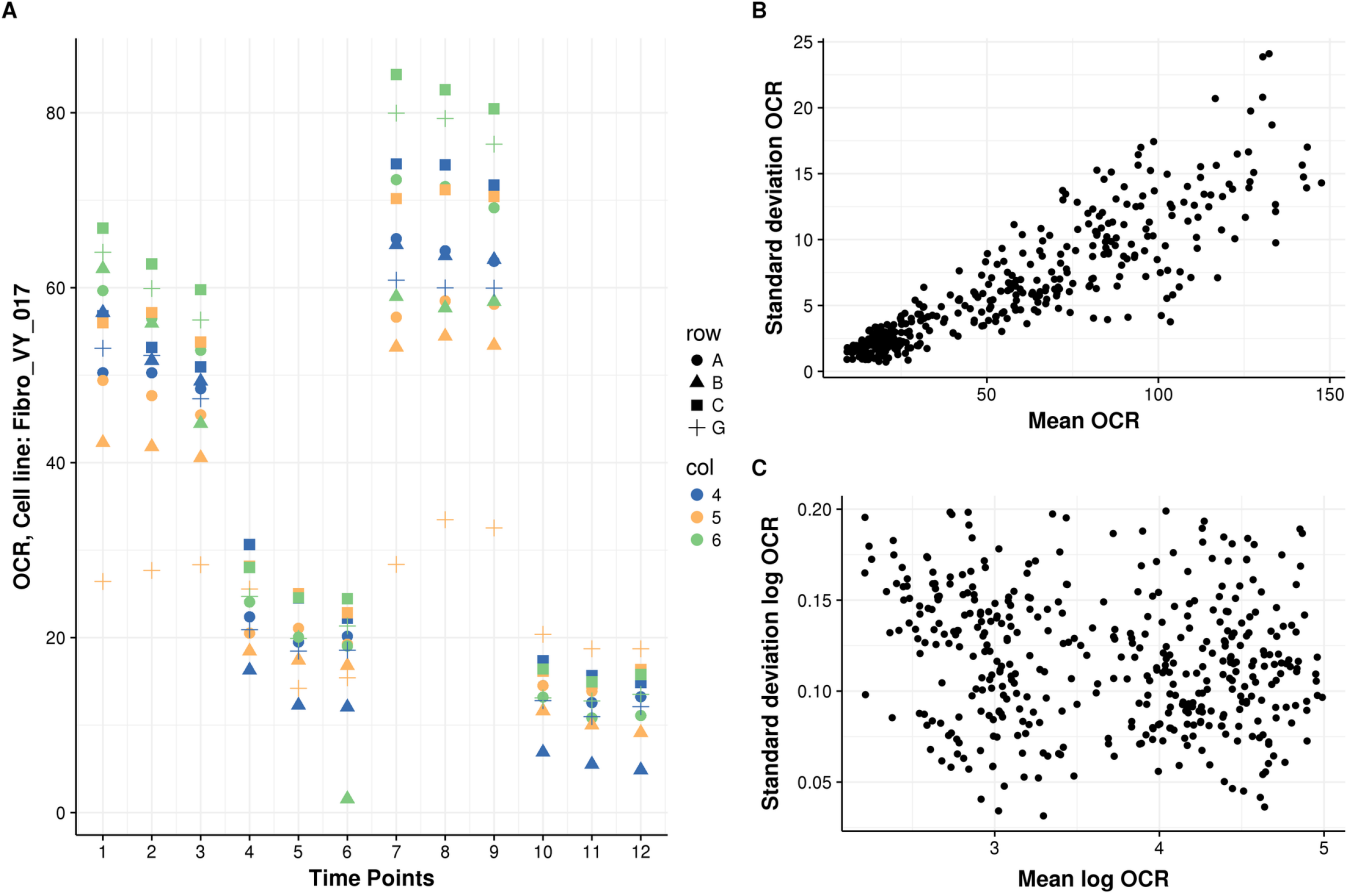
OCR behavior over time. (**A**) Typical time series replicates inside a plate. Behavior of OCR expressed in pmol/min (y-axis) of Fibro_VY_017 over time (x-axis). Colors indicate the row and shape the column of 12-well replicates. Variation increases for larger OCR values, OCR has a systematic well effect, and there are two types of outliers: well-level and single-point. (**B**) Scatterplot of standard deviation (y-axis) vs. mean (x-axis) of all three time replicates of each interval, well, and plate of OCR of NHDF only shows a positive correlation (*n* = 409). (**C**) The same as (B) but for the logarithm of OCR, where the correlation disappears.

Fig 2A shows representative replicate time series, with data from 12 wells for one biological sample in a single plate depicting commonly observed variations.

First, outlier data points occurred frequently. We distinguished two different types of outliers: entire series for a well (e.g., well G5 in Fig 2A) and individual data points (e.g., well B6 at time point 6 in Fig 2A). In the latter case, eliminating the entire series for well B6 would be too restrictive and would result in the loss of valuable data from the other 11 valid time points. Therefore, methods for detecting outliers taking these two possibilities into consideration must be devised.

Second, we noticed a proportional dependence of OCR value and standard deviation between replicates (Fig 2B), suggesting that the error is multiplicative. Unequal variance, or heteroscedasticity, can strongly affect the validity of statistical tests and the robustness of estimations. Therefore, we propose modeling OCR on a logarithmic scale, where the dependence between the variance and the mean disappears (Fig 2B and 2C). The activities of respiratory chain enzymes such as NADH-ubiquinone reductase also obey log-normal distributions [33].

Third, we observed systematic effects in OCR between wells (e.g., OCR values of well C6 are among the highest, while OCR values of well B5 are among the lowest at all the time points; Fig 2A). Variations in cell number, initial conditions, treatment concentrations, or fluorophore sleeve calibration can lead to systematic differences between wells, which we refer to as well effects. To investigate whether well effects could be corrected using cell number to a large extent as in [26], we counted the number of cells after the experiments using Cyquant (Materials and methods). As expected, the median OCR for each interval grows linearly with cell number measured at the end of the experiment (Spearman’s rho between 0.32 and 0.47, *P* < 2.2×10^−16^, S1A Fig). However, the relationship is not perfect, reflecting important additional sources of variations and also possible noise in measuring the cell number. Strikingly, dividing OCR by cell count led to a higher coefficient of variation (standard deviation divided by the mean) between the replicate wells than without that correction (S1B Fig). This analysis showed that normalization by the division of raw cell counts is insufficient and motivated us to derive another method to capture well effects. Finally, we found that sex does not significantly associate with OCR levels (S2 Fig), in agreement with [34].

### A statistical model for OCR within plates

Building on these insights, we introduced a statistical model for OCR within plates. For a given plate, we modeled the logarithm of OCR *y*_*w*,*t*_ of well *w* at time point *t* = 1,…,12 as a sum of time interval effects, well effects, and noise, that is:

$$y_{w,t}=\theta_{\mathrm{biosample}(w),\mathrm{interv}(t)}+\beta_{w}+\varepsilon_{w,t},$$ (1)

where *θ*_biosample(*w*),interv(*t*)_ is the time interval effect of the biological sample in well *w* for interval interv(*t*) = 1,…,4 of time point *t* (Fig 1A), *β*_*w*_ is the relative effect of well *w* compared to the reference well, and *ε*_*w*,*t*_ is the error.

We now present the OCR-Stats algorithm. For a given plate:

1. Fit the log linear model from Eq (1) using the least squares method, which consists of minimizing ∑_*w*_∑_*t*_(*y*_*w*,*t*_ − *θ*_biosample(*w*),interv(*t*)_ – *β*_*w*_)^2^, thus, obtaining the estimates ${\hat{\theta }}_{\mathrm{biosample}(w),\mathrm{interv}(t)}$ (which correspond to: *θ*_*I*_, *θ*_*Ai*_, *θ*_*M*_, *θ*_*Ei*_; Fig 1A) and ${\hat{\beta }}_{w}$.
2. For each well *w* and time point *t* in interval *i*, compute the log OCR well deviations: $d_{w,t}=y_{w,t}-{\hat{\theta }}_{\mathrm{biosample}(w),\mathrm{interv}(t)}-\frac{1}{n}\sum_{w}{\hat{\beta }}_{w}$, which is used to identify both the well and the single point outliers (Materials and methods, S3 Fig).
3. Identify and remove well level outliers (Materials and methods). Fit again, iteratively, until no more are found (S3A and S3B Fig).
4. Identify and remove single point outliers (Materials and methods). Fit again, iteratively, until no more are found (S3C and S3D Fig).
5. Compute the ratio-based metrics (Table 1), or scale back to natural scale in order to compute the bioenergetic measures [e.g.: $Basal\;respiration=\exp\left({\hat{\theta }}_{1}\right)-exp({\hat{\theta }}_{4})$].

Note that the well effect is modeled independently for each plate, that is, it corresponds to the effect of a well of a given plate and not to the effect of a well position shared across plates. We investigated whether there were positional effects and found that OCR measurements are lower in the edges by a median of up to 13.1% (S4A and S4B Fig). However, these positional effects were consistent across intervals (S4A and S4B Fig). Consequently, these positional effects are to a large extent canceled (S4C and S4D Fig) when using the metrics that we suggest (Table 1) because they involve differences of log OCRs. One exception was row A, where median differences of up to 2.2% were observed for the ETC-dependent OCR proportion and for the maximal over initial OCR fold change (S4C and S4D Fig). Practitioners could avoid all four edges and not only the four corners as typically done. However, these systematic deviations are small compared to the amplitude of biological effects typically investigated (not less than 12%–30% [30–32]). Altogether, this approach led to coefficients of variation between wells of the same plate of 11%, 14%, 13%, and 17% for intervals 1, 2, 3 and 4 respectively (Materials and methods).

### Variations between plates

After analyzing the OCR variation among the wells inside plates, we aimed to study the variation across multiple plates. Using data from the controls NHDF, we found that the variability between plates in all the intervals is much larger than that between wells (S2 Table and S5 Fig). Variations between plates can arise, for example, due to differences in temperature, seeding time, growth time, growth medium, or sensor cartridge [13]. Moreover, treatment efficiencies can also vary between plates, but independently from each other. For example, the concentration of rotenone may differ in one plate. That would affect the OCR measurements of all the wells in that plate, but only in time interval 4.

**Fig 3:**
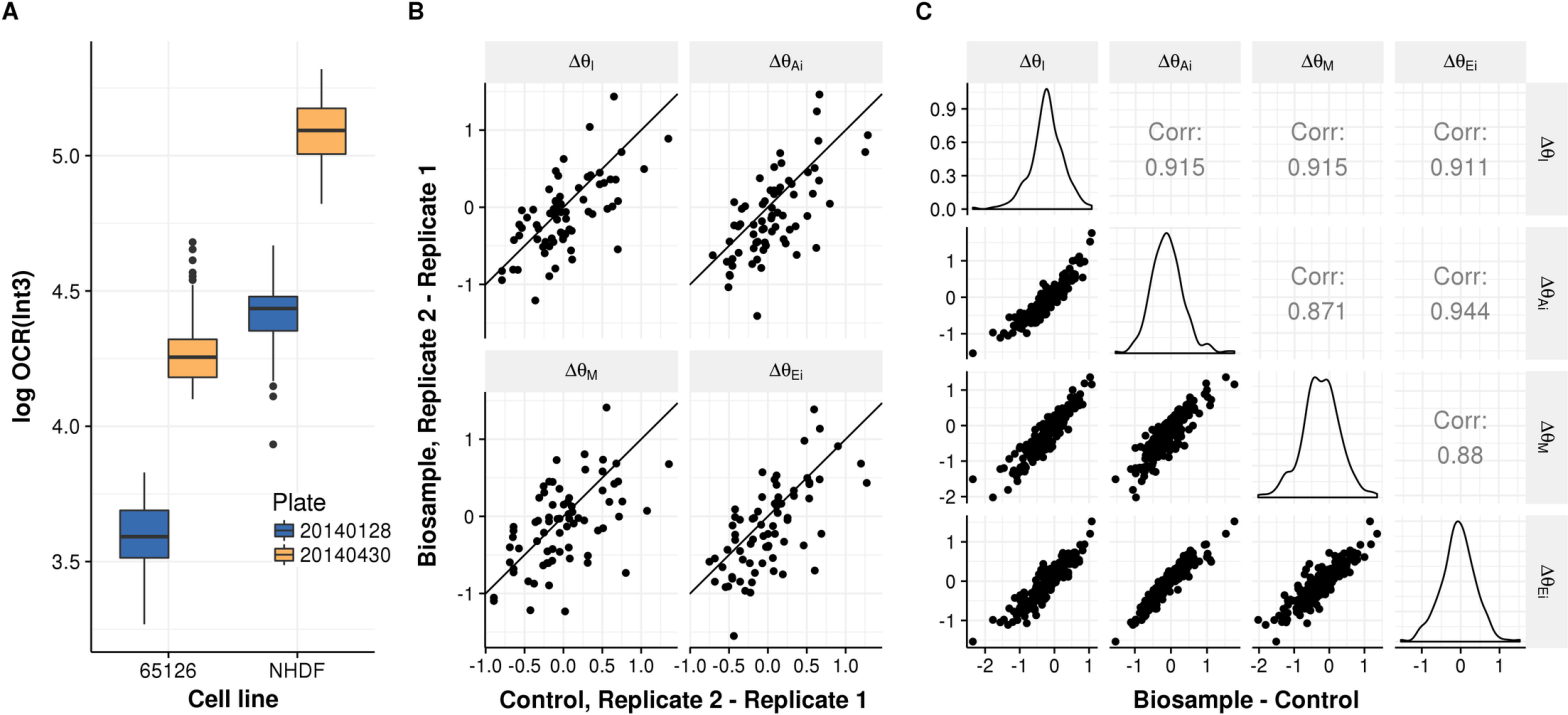
Plate-interval effect. (**A**) Log of OCR in interval 3 (y-axis) for the cell lines #65126 and NHDF (x-axis), which were seeded in two different plates (color-coded). The similar increase in OCR from plate #20140128 to #20140430 in both biological samples suggests that there is a systematic plate-interval effect. (**B**) Scatterplots of the differences of the logarithm of OCR levels Δ*θ* of all possible 2 by 2 combinations of repeated biological samples across experiments (y-axis) against their respective controls (NHDF) (x-axis) showing that there is a positive correlation (I_1_: *ρ* = 0.64, *P* < 2.3×10^−8^, I_2_: *ρ* = 0.65, *P* < 1.2×10^−8^, I_3_: *ρ* = 0.52, *P* < 1.2×10^−5^, I_4_: *ρ* = 0.64, *P* < 1.4×10^−8^), confirming a systematic plate-interval effect (*n* = 63). **(C)** Scatterplot of the difference of log OCR levels Δ*θ* of all the biological samples vs. their respective control (both axes) of every interval with respect to another. All the differences Δ*θ* correlate with each other even after removing the plate-interval effect (by subtracting control values).

Next, we investigated whether our assumption of systematic plate-interval effects held. We indeed observed a similar increase in OCR in interval 1 on both biological samples on plate #20140430 with respect to plate #20140428 (Fig 3A). To test whether this tendency held across the repeated biological samples, we compared all the replicate pairings with their respective NHDF controls and found a positive correlation in all the time intervals (Fig 3B), suggesting a plate-interval effect. These observations show the importance of basing conclusions from observations across multiple plates and for seeding a control cell line on every plate.

### Statistical testing for the comparison of biological samples across plates

We then set up to devise a model to statistically assess difference in OCR ratios between two biological samples across multiple plates. Since there is a remaining systematic effect across intervals at the plate level (Fig 3C) and because of the plate-interval effects, we recommend using ratios of OCR levels (i.e. differences in the logarithmic scale) (Table 2).

**Table 2:** OCR ratio-based differences for statistical testing.

OCR ratios	Tested differences ΔΔ*θ*
E/I-proportion	(*θ*_*I*,*b*_ – *θ*_*Ei*,*b*_) – (*θ*_*I*,*c*_ – *θ*_*Ei*,*c*_)
A/I-proportion	(*θ*_*I*,*b*_ – *θ*_*Ai*,*b*_) – (*θ*_*I*,*c*_ – *θ*_*Ai*,*c*_)
E/Ai-proportion	(*θ*_*Ai*,*b*_ – *θ*_*Ei*,*b*_) – (*θ*_*Ai*,*c*_ – *θ*_*Ei*,*c*_)
M/I-fold change	(*θ*_*M*,*b*_ – *θ*_*I*,*b*_) – (*θ*_*M*,*c*_ – *θ*_*I*,*c*_)
M/Ei-fold change	(*θ*_*M*,*b*_ – *θ*_*Ei*,*b*_) – (*θ*_*M*,*c*_ – *θ*_*Ei*,*c*_)

For each OCR ratio from Table 1, we present the differences ΔΔ*θ* to be used when testing a biological sample *b* against a control *c* on each plate.

**Fig 4:**
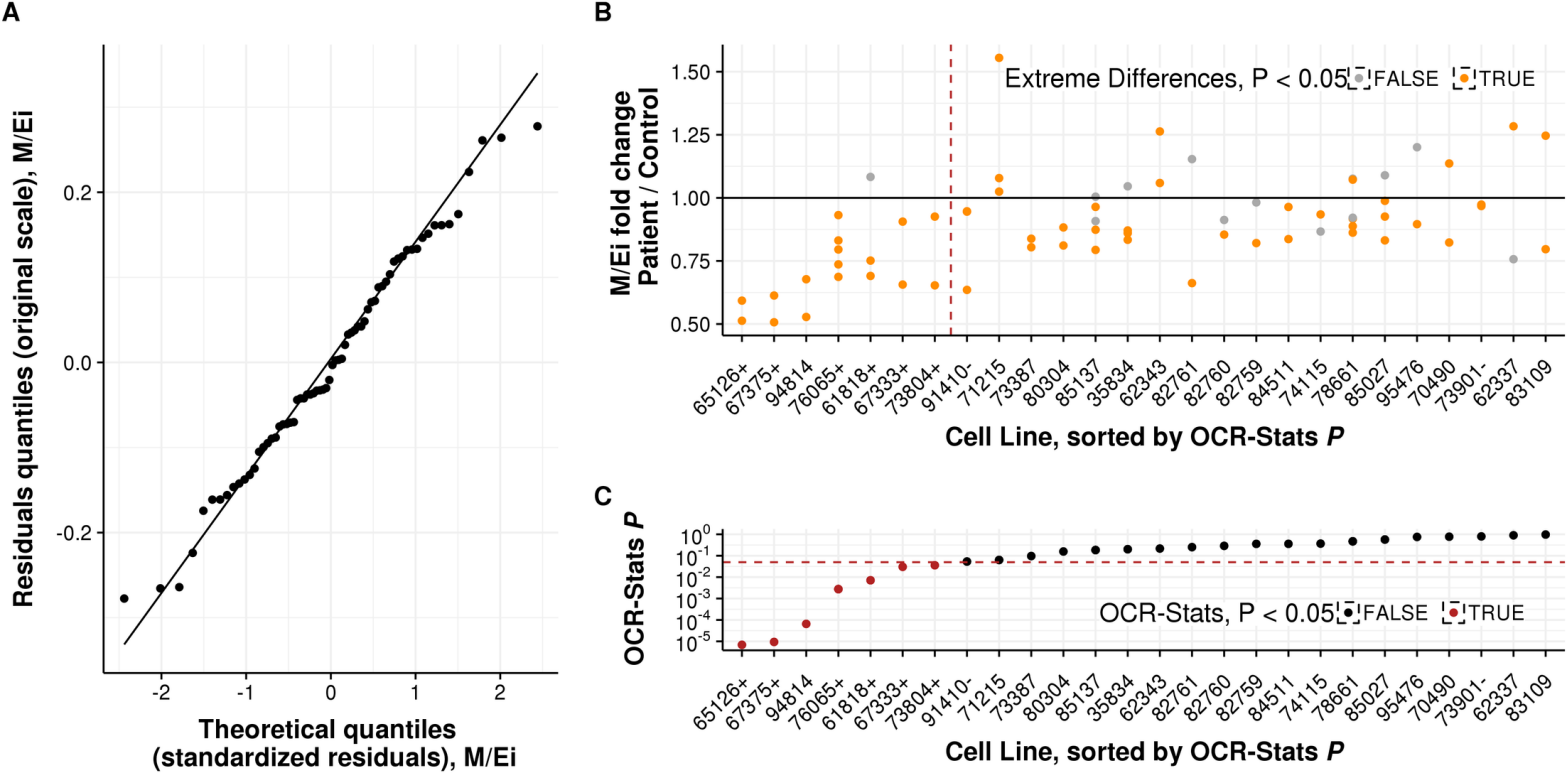
Statistical testing of M/Ei-fold change patient vs. control on multiple plates. **(A)** Ratio of M/Ei-fold change (y-axis) of all the cell lines repeated across plates (x-axis) and their respective controls, sorted by the p-value obtained using the OCR-Stats method. Left of the red dashed line are cell lines with significantly lower M/Ei-fold change using OCR-Stats. Dots in orange represent biological samples with significantly lower or higher M/Ei-fold change using the ED method. Highlighted positive (+) and negative (–) controls. **(B)** Similar to (A), but depicting the p-value in logarithmic scale (y-axis) using OCR-Stats. Red dashed line at *P* = 0.05. Dots in red represent cell lines with significantly lower M/Ei-fold change using the OCR-Stats method. **(C)** Quantile-quantile theoretical (x-axis) vs. observed (y-axis) plot of the residuals *ϵ*_*b*,*p*_ of the linear model (2) applied to M/Ei-fold change. Points are lying on the diagonal as expected from normally distributed residuals.

Subsequently, for any given OCR ratio (e.g., M/Ei-fold change, Tables 1 and 2), we test the differences of the OCR log-ratios ΔΔ*θ* of a biological sample *b* versus a control *c* using the following linear model:

$${\Delta \Delta \theta }_{b,p}=\mu_{b}+\epsilon_{b,p},$$ (2)

where ΔΔ*θ*_*b*,*p*_ is one OCR log-ratio difference of interest (Table 2) inside a plate *p*. We fit this model over our complete dataset using linear regression, thus obtaining one value ${\hat{\mu }}_{b}$ per OCR ratio and biological sample *b*. Then, we tested these against the null hypothesis *μ*_*b*_ = 0 to compute p-values and confidence intervals (Materials and methods). Fitting the linear model of Equation (Eq 2) over the complete dataset gives a robust estimate of the standard deviation of the error term. Applying this approach, we found no evidence against the normality and homoscedasticity assumption of OCR-Stats as the quantile-quantile plots of the residuals aligned well along the diagonal (Fig 4A and S6 Fig).

### Benchmark

We applied OCR-Stats statistical testing, Extreme Differences plus Wilcoxon test within each plate (within-plate ED), and Extreme Differences plus Wilcoxon test across plates (across-plate ED) to obtain the M/Ei-fold change and maximal respiration (MR) of all of the 26 cell lines that were seeded in more than one plate (Materials and methods). For every approach, we computed p-values for significant fold changes against the controls. Six of these cell lines are derived from patients with rare variants in genes associated with an established cellular respiratory defect, allowing the assessment of the sensitivity (or statistical power) of each approach (S3 Table, [35–39]). Additionally, two cell lines (#73901 and #91410) repeatedly showed no significant respiratory defects in earlier studies and served as negative controls [40,41].

The within-plate ED method reported significantly higher or lower MR for 56 out of 69 (81.2%) biological samples with respect to the control (Fig 4B and S3 Table). Moreover, the within-plate ED method reported one or more significant differences for all the 26 cell lines, and one or more non-significant differences for 11 cell lines (Fig 4B). For two cell lines, the within-plate ED method returned significant differences with opposite signs (cell lines #78661, #83109, Fig 4B). These ambiguous results show the importance of testing using multiple plates and suggest the need for a more robust approach than the within-plate ED. One approach to evaluate samples measured in multiple plates is to perform a Wilcoxon test on the ED values averaged per plate (across-plate ED, Materials and methods). However, this requires at least five plate replicates in order to obtain significant results. Here, one cell line only, #78661, was found to have significantly impaired OCR in this way. For these data, OCR-Stats was much more conservative than within-plate ED and found only 7 out of 26 (26.9%) cell lines to have aggregated significantly lower M/Ei-fold change than the control, including all six positive control cell lines (Fig 4B and 4C, and S3 Table). Moreover, OCR-Stats did not report significant M/Ei-fold changes for the two negative controls.

**Fig 5:**
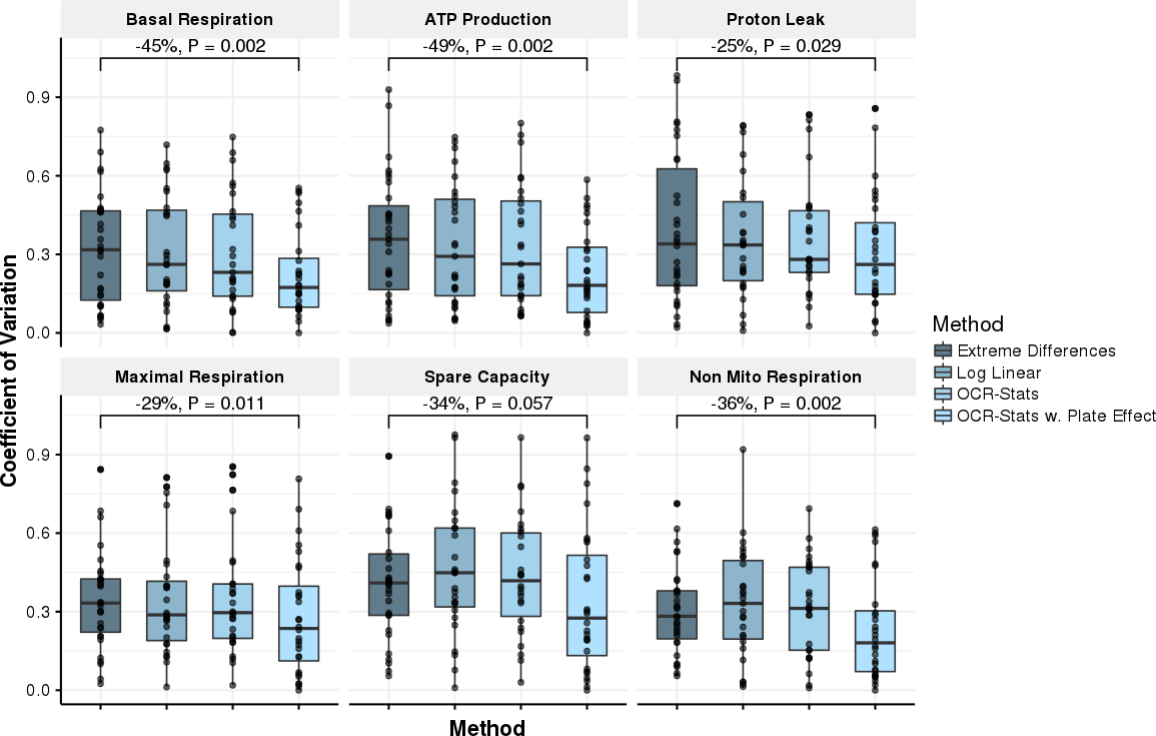
Benchmark using coefficient of variation. Coefficient of variation (CV = standard deviation/mean, y-axis) of replicates across experiments (*n* = 26) using different methods (x-axis) to estimate the six bioenergetic measures. In all, except for Spare Capacity, OCR-Stats with plate-interval effect showed significantly lower variation with respect to the Extreme Differences method. P-values obtained from one-sided paired Wilcoxon test.

Furthermore, we computed the coefficient of variation (standard deviation divided by mean) of the six bioenergetic measures in the natural scale (Table 1) of all the repeated biological samples across plates for the following methods: i) the default Extreme Differences (ED) method (Materials and methods) provided by the vendor, ii) the log linear (LL) corresponding to steps 1 and 2 of the OCR-Stats algorithm, iii) complete OCR-Stats (LL + outlier removal), and iv) OCR-Stats after correcting for plate effect (OCR-PE) using Eq (4) (Materials and methods). Each step contributed to a decrease in the coefficient of variation, obtaining a final significant reductions of 45% and 29% in basal and maximal respiration, respectively, from plate-corrected OCR-Stats (OCR-PE) with respect to ED (*P* < 0.012, one-sided Wilcoxon test) (Fig 5). Taken together, these results show that OCR-Stats successfully identifies and decreases the variation within and between plates, providing more stable testing results, which translates into fewer false positives.

### Power analysis

**Fig 6:**
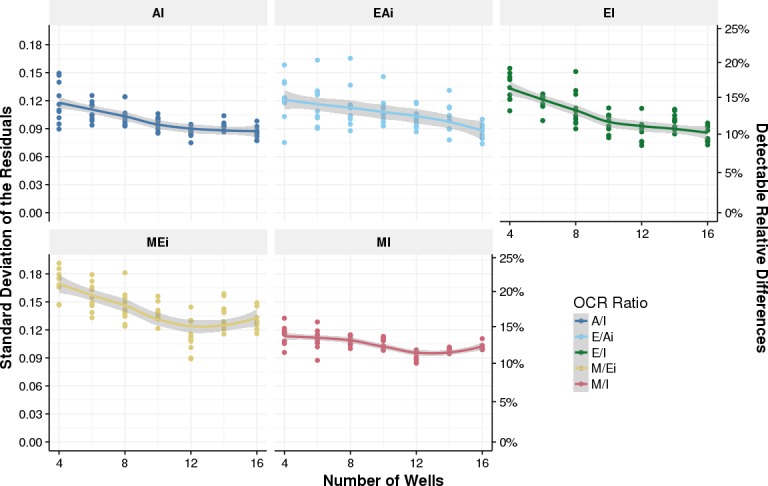
Power analysis. Standard deviation of the residuals from the model in Eq (2) (left y-axis) against number of wells per biological sample and per plate (x-axis) for each OCR log-ratio difference (Table 1). The right y-axis corresponds to the minimal detectable relative differences using three plates at 5% significance level (Materials and methods). For every number of wells, the 10 data points correspond to each of the 10 random samplings without replacement of the wells per biological sample and per plate.

Finally, we investigated the statistical power of OCR-Stats in this dataset. Specifically, we are interested in determining the minimum relative differences our method is able to significantly detect, and the minimal number of well replicates needed. We subsetted the number of wells of the repeated biological samples to 4, 6, 8, 10, 12, 14, and 16 wells on each plate, and used the OCR-Stats algorithm (including outlier removal) and statistical testing to obtain the residuals *ϵ*_*b*,*p*_ and their standard deviation (Fig 6). Assuming three plates per comparison and 16 wells per plate, these standard deviations allow detecting relative differences of 10% to 15% depending on the considered log OCR ratios differences for significance level of 5% (Fig 6, right y-axis, Materials and methods). Relative differences of 10% to 15% are in line with reported detected variations in the literature which we found to be as low as 12%-30% [30–32]. This analysis also suggests to seed at least 12 wells per biological sample per plate, since we observed increased standard deviations of the residuals for numbers of wells smaller than 12. Note that this power calculation is based on measurements performed in our laboratory only. Other laboratories might have larger or smaller measurement variations. Nonetheless, our procedure could be used as a guideline for power calculation.

## Discussion and conclusion

Mitochondrial studies using extracellular fluxes, specifically the XF Analyzer from Seahorse, are gaining popularity and are finding their way into diagnostics; therefore, it is of paramount importance to have an appropriate statistical method to estimate the OCR levels from the raw data. Here, we have developed such a model, the OCR-Stats algorithm, which includes approaches to control for well and plate-interval effects, and automatic outlier identification.

We found that dividing cellular OCR by cell number involved the introduction of more noise than was seen for uncorrected data. Here, we always seeded the same number of cells. Hence, the variations across wells that we observed in the cell number at the end of the experiments are largely overestimated by noise in the measurements. In other experimental settings in which different numbers of cells are seeded, we suggest the inclusion of an offset term to the model in Eq (1) equal to the logarithm of the seeded cell number to control for this variation by design. In addition, the Seahorse XF Analyzer can be used on isolated mitochondria and on isolated enzymes, where a normalization approach is to divide OCR by mitochondrial proteins or enzyme concentration [42]. However, as described here for cellular assays, robust normalization procedures require careful analysis.

We demonstrated that OCR comparisons should be performed using ratios rather than using differences, and that the cell lines must be seeded on the same plate, as this eliminates sources of variation like cell number, and well positional and plate-interval effects. We introduced a linear model, the OCR-Stats statistical testing, and showed that the results agree with previous results of patients diagnosed with mitochondrial disorders. We showed that the variation in differences of OCR log-ratios ΔΔ*θ*_*b*,*p*_ for the same biological sample across plates is large, and that, consequently, samples should be seeded in multiple plates. Note that a contaminated sample can increase the variability, affecting the significance of all the other samples. Therefore, it is important to detect such samples and to exclude them from further analysis. By doing power analysis, we showed that our method is able to detect relative differences of 10% - 15%, and that the minimum number of well replicates per biological sample in a 96-well plate should be 12.

We encourage users to consider all five metrics (Table 1). Severely affected cell lines with strongly reduced E/I-proportion might not necessarily show a clear effect on M/Ei-fold change. For example, cell line #73387 was found to have a lower, but not significantly (*P* < 0.10), M/Ei-fold change (the most common metric used throughout the literature, Fig 4C and S3 Table), but when analyzing its E/I proportion, we found that it was significantly lower than the control (*P* < 1.2×10^−7^). This result is consistent with its genetic diagnosis, a homozygous loss of function variant in the *PET100* gene, which is involved in biogenesis of mitochondrial complex IV (S1 Table, [43]).

In principle, OCR-Stats should be able to estimate ECAR levels. To guarantee that the method is indeed applicable, similar analyses as performed here should be done beforehand. Preliminary investigations suggest that the nature of noise (outliers, multiplicative) is similar to that for OCR.

## Materials and methods

### Biological material

All the biological samples were derived from primary fibroblast cell lines of humans suffering from rare mitochondrial diseases, established in the framework of the German and European networks for mitochondrial disorders mitoNet and GENOMIT. All the individuals or their guardians provided written informed consent for their cell lines to be used for evaluation and testing, in agreement with the Declaration of Helsinki and approved by the ethical committees of the centers participating in this study. All the assays were performed in accordance with the local approval of the ethical committee of the Technical University of Munich. The controls are primary patient fibroblast cell lines, normal human dermal fibroblasts (NHDF) from neonatal tissue, commercially available from Lonza, Basel, Switzerland.

### Measure of extracellular fluxes using Seahorse XF96

We seeded 20,000 fibroblast cells in each well of a XF 96-well cell culture microplate in 80 ml of culture medium, and incubated them overnight at 37°C in 5% CO_2_. The four corners were left only with medium for background correction. The culture medium was replaced with 180 ml of bicarbonate-free DMEM and cells were incubated at 37°C for 30 min before measurement. Oxygen consumption rates (OCR) were measured using an XF96 Extracellular Flux Analyzer [20]. OCR was determined at four levels: with no additions, and after adding oligomycin (1 μM), carbonyl cyanide 4-(trifluoromethoxy) phenylhydrazone (FCCP, 0.4 μM), and rotenone (2 μM) (additives purchased from Sigma at highest available quality). After each assay, manual inspection was performed on all wells using a conventional light microscope. The wells for which the median OCR level did not follow the expected order, namely, median[OCR(Int_3_)] > median[OCR(Int_1_)] > median[OCR(Int_2_)] > median[OCR(Int_4_)] (Fig 1A), were discarded (977 wells, 10.47%). It is important to note that other cell lines, or cell lines under certain conditions, may not react as expected to the standard treatments; therefore, they should not be discarded. In addition, we excluded contaminated wells and wells in which the cells got detached (461 wells, 4.94%) from the analysis. All the raw OCR data are available in S4 Table.

### Cell number quantification

The cell number was quantified using the CyQuant Cell Proliferation Kit (Thermo Fisher Scientific, Waltham, MA, USA), according to the manufacturer’s protocol. In brief, the cells were washed with 200 *μ*L PBS per well and frozen in the microplate at -80°C to ensure subsequent cell lysis. The cells were thawed and resuspended vigorously in 200 *μ*L of 1x cell-lysis buffer supplemented with 1x CyQUANT GR dye per well. The resuspended cells were incubated in the dark for 5 min at RT, whereupon fluorescence was measured (excitation: 480 nm, emission: 520 nm).

### Extreme differences (default) method to compute bioenergetic measures

On every plate independently, for each well, in interval 1 take the OCR corresponding to the last measurement, in intervals 2 and 4 take the minimum, and in interval 3 the maximum OCR value [19]. Then, use the corresponding differences to estimate the bioenergetic measures. Report the results per patient as the mean across wells plus standard deviation or standard error, separately for each plate.

### Outlier removal

For each sample *s* and well *w*, compute the mean across time points of its squared deviations: $s_{w}≔{mean}_{t}(d_{w,t}^{2})$, thus, obtaining a vector **s**. Identify as outliers the wells whose *s*_*w*_ > median(**s**) + 5 mad(**s**), where mad, median absolute deviation, is a robust estimation of the standard deviation (S3A Fig). We found that deviations by 5 mad from the median were sufficiently selective in practice. Compute the vector of estimates $\hat{\theta }$ using the remaining wells and iterate this procedure until no more wells are identified as outliers. It required eight iterations until convergence and around 16.5% of all the wells were found to be outliers (S3B Fig).

Single point outliers are identified in a similar way. After discarding the wells that were found to be outliers in the previous step, categorize as outliers single data points whose $d_{w,t}^{2}>{median}_{t}(d_{w,t}^{2})+7\;{mad}_{t}(d_{w,t}^{2})$ (S3C Fig). Iterate until no more outliers are found. It required 19 iterations until convergence and approximately 6.1% of single points were found to be outliers (S3D Fig).

### Coefficient of variation between wells of the same plate

Using only the controls NHDF, we computed the standard deviation *σ*_*p*,*i*_ of the logarithm of OCR across all the wells for each plate *p* and interval *i*. Then, we computed the median across plates, thus, obtaining one value ${\overset{-}{\sigma }}_{i}$ per interval (${\overset{-}{\sigma }}_{1}=0.10,{\overset{-}{\sigma }}_{2}=0.13,{\overset{-}{\sigma }}_{3}=0.12,{\overset{-}{\sigma }}_{4}=0.16$). Coefficients of variation in the natural scale were approximated by taking the exponential of these standard deviations.

### OCR-Stats statistical testing

We fitted Eq (2) using linear regression as implemented in the base R function lm(). P-values for each ratio against the null hypothesis *μ*_*b*_ = 0 are obtained with the default test (Student’s t-test) returned by the summary function on the lm fit object.

### Power calculation of multi-plate experiments

Minimal detectable effects for OCR-ratio based metrics (Table 2) at 95% confidence level were estimated using the following equation:

$$\exp\left(1.96\frac{\mathrm{sd}(\epsilon_{b,p})}{\sqrt{n}}\right)-1,$$ (3)

where *ϵ*_*b*,*p*_ are the residuals from Eq 3 and 1.96 corresponds to the approximate value of the 97.5 percentile of the standard normal distribution. We obtain the metrics on Table 2 by setting *n* = 3.

### Plate-interval effect benchmark

For benchmarking, we correct for the plate-interval effect using only the data from the controls NHDF *c* of each plate using the following model:

$$y_{control,t,p}=\theta_{control,\mathrm{interv}(t)}+\beta_{\mathrm{interv}(t),p}+\varepsilon_{t,p}.$$ (4)

We solved it using the least squares method and used the effects ${\hat{\beta }}_{i,p}$ as offsets in Eq (1). We recomputed ${\hat{\theta }}_{b,i}$ values accordingly and scaled back to the natural scale to calculate the bioenergetic measures and the coefficient of variation of all repeated the biological samples, except the control (Fig 5).

### Multi-plate averaging method

In the case of inter-plate comparisons, the multi-plate averaging method takes the mean and standard error of the bioenergetic measures obtained using the Extreme Differences (ED) method of all the repeated biological samples across plates [44].

## Supporting information

S1 Table Sample metadata. Each row corresponds to a different fibroblast cell line (Fibroblast id) from a patient with a mitochondrial disorder. In cases in which the causal pathogenic gene was found and validated, it appears in the Gene column with its respective OMIM number. N indicates the number of experiments of each sample.
(TXT)

S2 Table Coefficient of variation within and between plates. Coefficient of variation computed as mean/standard deviation of OCR within and between plates using the controls NHDF only, in each time interval.
(TXT)

S3 Table M/Ei-fold change and maximal respiration (MR) differences of samples repeated across experiments with respective p-values. Each row corresponds to a different biological sample (cell_culture), with the difference in M/Ei-fold change and MR with respect to the control NHDF, in single and multiple plates. P-values computed using OCR-Stats and ED method within and between plates.
(TXT)

S4 Table OCR raw data from all experiments. OCR and cell number raw data for each of the 126 plates and 203 samples across all the 12 time points and 4 treatments.
(TXT)

S1 Fig Normalizing by cell number does not reduce variation. **(A)** OCR per well median (y-axis) vs. cell number (in thousands, x-axis) of the controls NHDF in all experiments (*n* = 2,192 for each panel) showing that there is a positive correlation in all the time intervals (I_1_: *ρ* = 0.47, I_2_: *ρ* = 0.45, I_3_: *ρ* = 0.40, I_4_: *ρ* = 0.33; *P* < 2.2×10^−16^ for all the intervals). **(B)** Coefficient of variation (y-axis) of well replicates within plates for raw OCR and OCR normalized dividing by cell count (x-axis), split for each time interval. Each point represents a different sample. In all the four intervals, not only did normalization not reduce the coefficient of variation, but it actually increased it. P-values obtained from two-tailed Wilcoxon tests.
(PNG)

S2 Fig OCR does not depend on sex. OCR levels **θ** (y-axis) split by sex (x-axis). We see no significant difference in any time interval (*n* = 45 male, 30 female).
(PNG)

S3 Fig Outlier detection. **(A)** Number of wells (y-axis) identified as outliers on each iteration (x-axis). Around 16.5% of all valid wells detected as outliers. **(B)** Mean (per well) squared errors distribution for cell line Fibro_VY_014. Wells beyond the red line (median + 5×mad) are recognized as well-level outliers. **(C)** Number of single-point outliers (y-axis) identified on each iteration (x-axis). Around 6.1% of remaining data (after removing well outliers) detected as single point outliers. **(D)** Squared error distribution for cell line Fibro_VY_076. Points beyond the red dashed line (median + 7×mad) are recognized as single-point outliers.
(PNG)

S4 FigInvestigation of location effect.**(A)** Deviations of the log OCR measurements with respect to the interval effect ($y-\hat{\theta }$, y-axis) behavior across rows (x-axis). In general, a tendency for higher OCR is observed on the center of the plate across all time intervals. **(B)** The same as (A) but for columns (x-axis). Lower values observed in the edges. **(C, D)** Well-level OCR ratio subtracted interval level OCR ratio (Table 1) across rows (x-axis, C) and columns (x-axis, D). All the location effects get canceled, except for row A where it remains relatively low.(PNG)

S5 Fig Variation between plates is larger than variation within plates. Boxplot showing OCR in time interval 3 (x-axis) of NHDF seeded in 10 randomly selected plates (y-axis) reflecting that the variation between is larger than the variation within plates. Red line: mean of OCR across all plates. This trend was observed across all the plates and for all the intervals (S2 Table).
(PNG)

S6 Fig Residuals from the linear regression are consistent with a normal distribution. Quantile-quantile theoretical (x-axis) vs. observed (y-axis) plots of the residuals *ϵ*_*b*,*p*_ of the linear model from Eq (2). Points lie on the diagonal as expected from normally distributed residuals.
(PNG)
