# Supplementary figures and images for: OCR-Stats: Robust estimation and statistical testing of mitochondrial respiration activities using Seahorse XF Analyzer
Source: PLoS One. 2018 Jul 11;13(7):e0199938. doi: 10.1371/journal.pone.0199938 (PMC6040740; doi:10.1371/journal.pone.0199938)

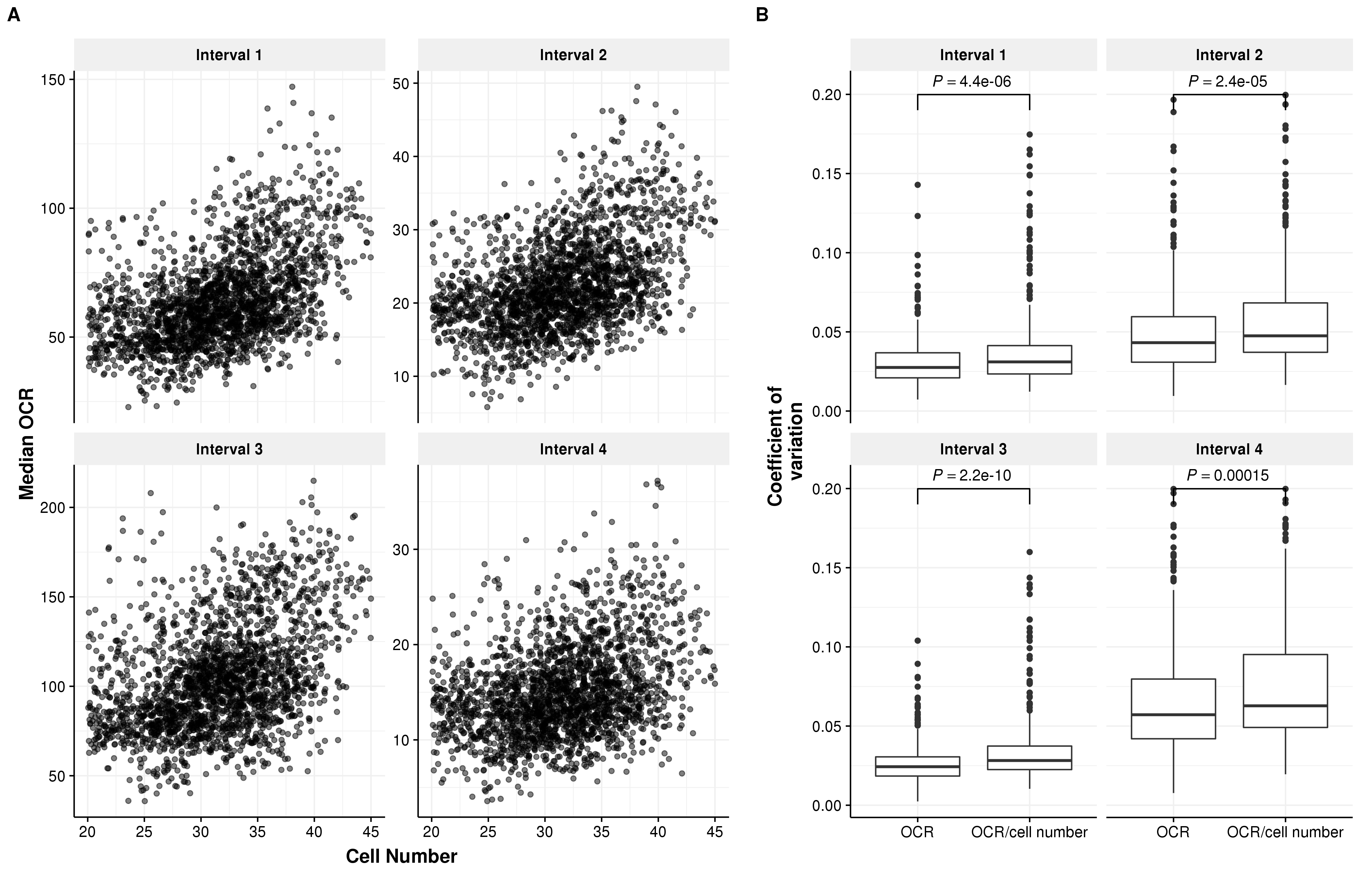

Supplement: S1 Fig — (A) OCR per well median (y-axis) vs. cell number (in thousands, x-axis) of the controls NHDF in all experiments (n = 2,192 for each panel) showing that there is a positive correlation in all the time intervals (I1: ρ = 0.47, I2: ρ = 0.45, I3: ρ = 0.40, I4: ρ = 0.33; P < 2.2×10^−16^ for all the intervals). (B) Coefficient of variation (y-axis) of well replicates within plates for raw OCR and OCR normalized dividing by cell count (x-axis), split for each time interval. Each point represents a different sample. In all the four intervals, not only did normalization not reduce the coefficient of variation, but it actually increased it. P-values obtained from two-tailed Wilcoxon tests. (PNG) [file pone.0199938.s005.png]

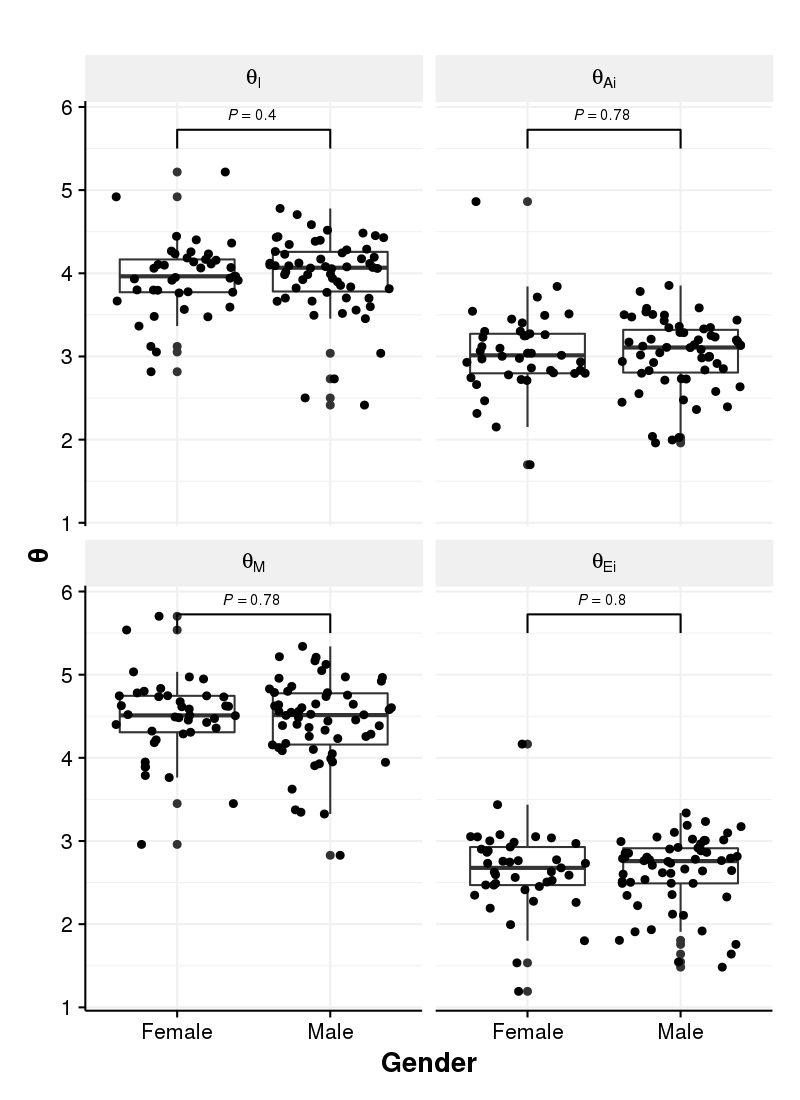

Supplement: S2 Fig — OCR levels θ (y-axis) split by sex (x-axis). We see no significant difference in any time interval (n = 45 male, 30 female). (PNG) [file pone.0199938.s006.png]

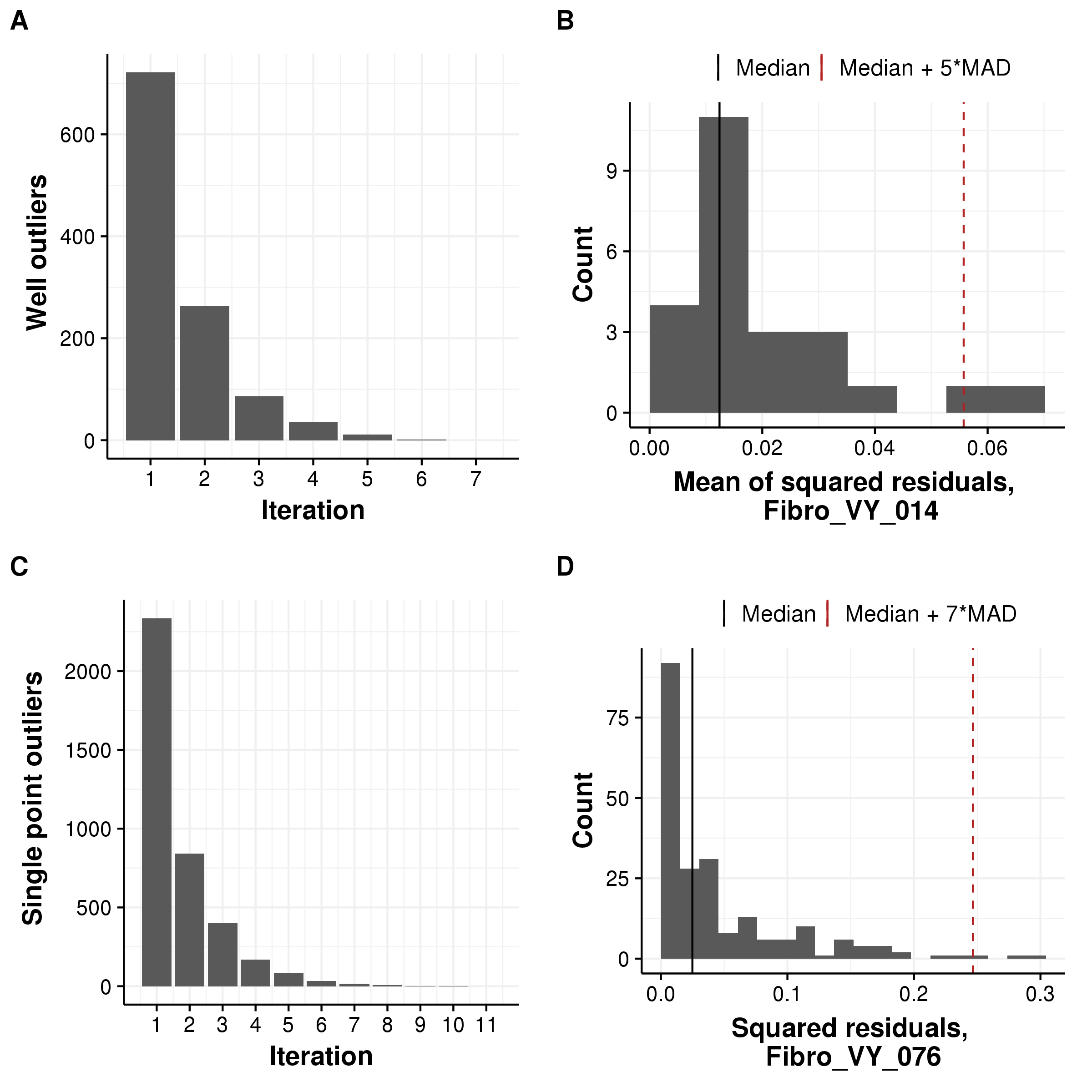

Supplement: S3 Fig — (A) Number of wells (y-axis) identified as outliers on each iteration (x-axis). Around 16.5% of all valid wells detected as outliers. (B) Mean (per well) squared errors distribution for cell line Fibro_VY_014. Wells beyond the red line (median + 5×mad) are recognized as well-level outliers. (C) Number of single-point outliers (y-axis) identified on each iteration (x-axis). Around 6.1% of remaining data (after removing well outliers) detected as single point outliers. (D) Squared error distribution for cell line Fibro_VY_076. Points beyond the red dashed line (median + 7×mad) are recognized as single-point outliers. (PNG) [file pone.0199938.s007.png]

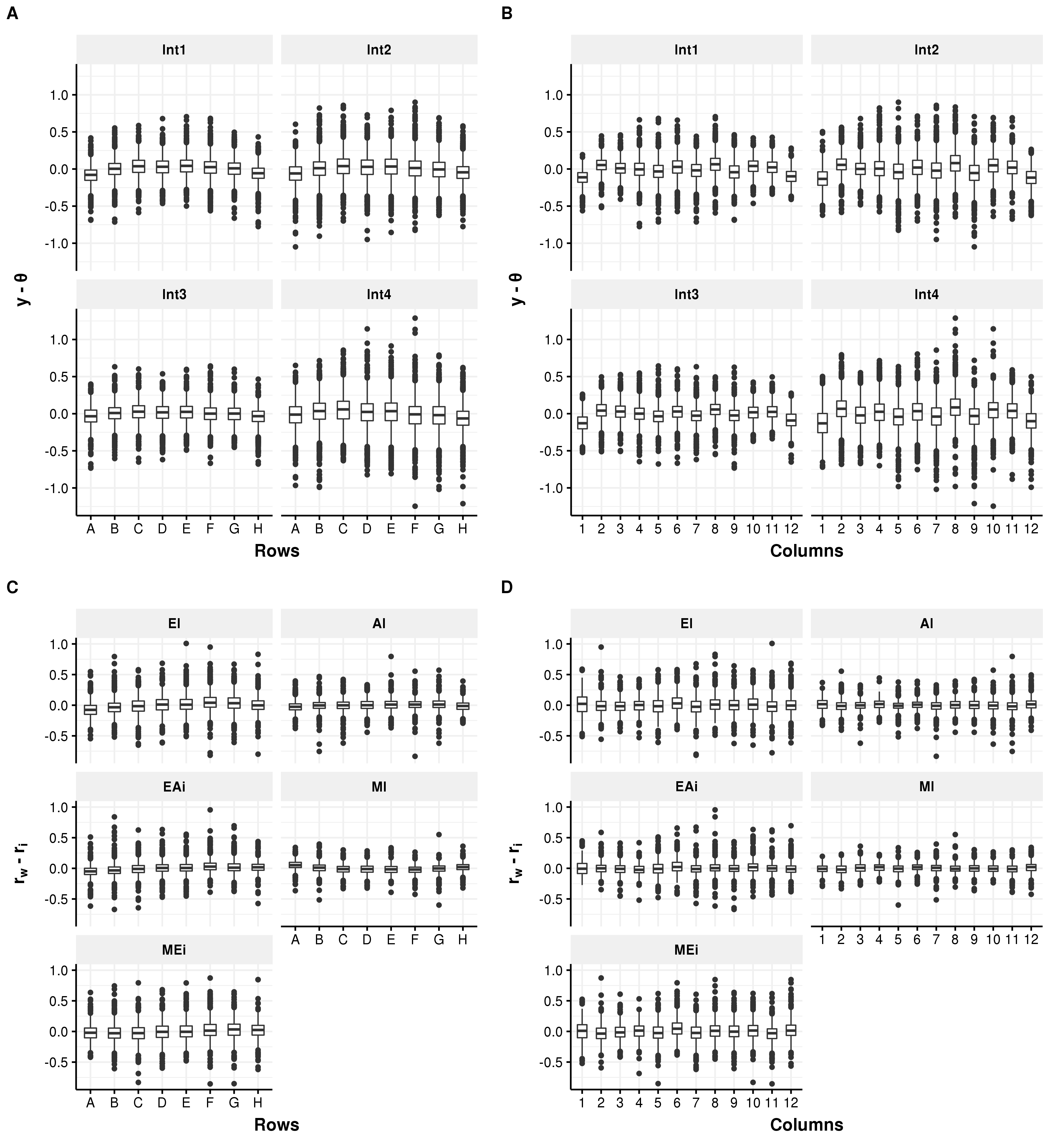

Supplement: S4 Fig — (A) Deviations of the log OCR measurements with respect to the interval effect (y−θ^, y-axis) behavior across rows (x-axis). In general, a tendency for higher OCR is observed on the center of the plate across all time intervals. (B) The same as (A) but for columns (x-axis). Lower values observed in the edges. (C, D) Well-level OCR ratio subtracted interval level OCR ratio (Table 1) across rows (x-axis, C) and columns (x-axis, D). All the location effects get canceled, except for row A where it remains relatively low. (PNG) [file pone.0199938.s008.png]

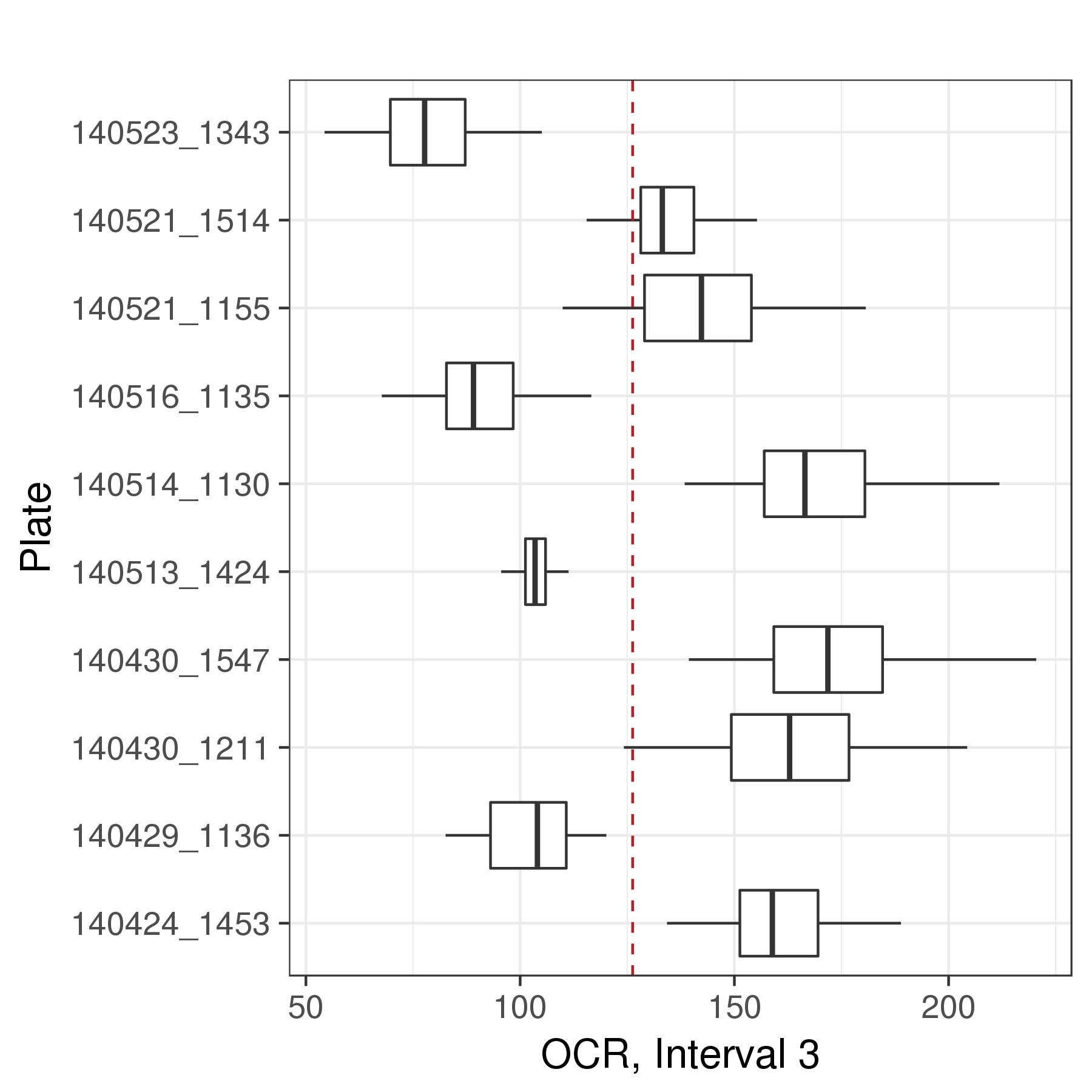

Supplement: S5 Fig — Boxplot showing OCR in time interval 3 (x-axis) of NHDF seeded in 10 randomly selected plates (y-axis) reflecting that the variation between is larger than the variation within plates. Red line: mean of OCR across all plates. This trend was observed across all the plates and for all the intervals (S2 Table). (PNG) [file pone.0199938.s009.png]

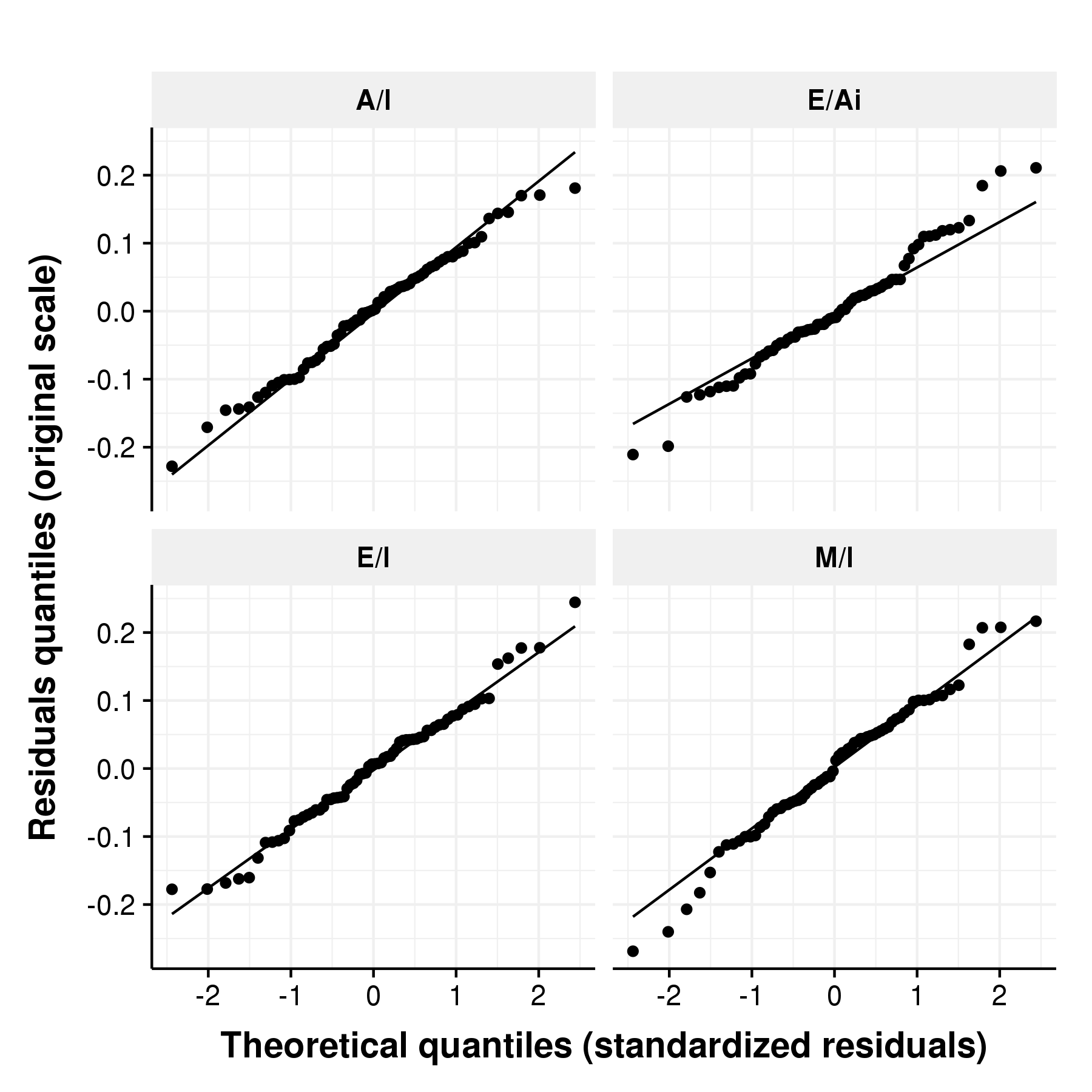

Supplement: S6 Fig — Quantile-quantile theoretical (x-axis) vs. observed (y-axis) plots of the residuals ϵb,p of the linear model from Eq (2). Points lie on the diagonal as expected from normally distributed residuals. (PNG) [file pone.0199938.s010.png]
